# Could the New Body Shape Index Predict the New Onset of Diabetes Mellitus in the Chinese Population?

**DOI:** 10.1371/journal.pone.0050573

**Published:** 2013-01-30

**Authors:** Sen He, Xiaoping Chen

**Affiliations:** Department of Cardiovascular Medicine, West China Hospital, Sichuan University, Chengdu, China; Consiglio Nazionale delle Ricerche, Italy

## Abstract

**Background:**

Anthropometric measures could predict the new onset of diabetes mellitus (DM). Recently, a new anthropometric measure (a body shape index, ABSI) was developed, and ABSI could predict premature mortality, even better than WC and BMI. The main aims of the study were to assess the DM predictive power of ABSI based on the follow-up data over 15 years collected from a general Chinese population.

**Methods and Findings:**

The data were collected in 1992 and then again in 2007 from the same group of 711 individuals. Because 24 of them were diagnosed with DM in 1992, our analysis was eventually based on the usable data collected from the remaining 687 individuals (male: 58.1%). ABSI was defined as WC/(BMI^2/3^height^1/2^), expressing WC and height in m. After adjusting for confounders, increasing the population ABSI by one standard deviation would result in an increased HR of 41% (95%CI: 12%–77%), compared to 73% (95%CI: 37%–118%) for BMI and 96% (95%CI: 53%–150%) for WC. WC had the best discriminatory power for DM (AROC: 0.701, 95%CI: 0.641–0.760), followed by BMI (AROC: 0.668, 95%CI: 0.601–0.734) and ABSI (AROC: 0.647, 95%CI: 0.581–0.713).

**Conclusions:**

ABSI could predict the new onset of DM in the Chinese population independently. Since the confidence intervals for the HR coefficients and AROCs overlapped, the predictive ability of ABSI for DM was not better than WC and BMI. The three measures had similar predictive abilities for DM. Further studies about ethnic specificities of ABSI are needed and warranted.

## Introduction

There is no cure for diabetes mellitus (DM); thus, prevention is the best intervention. Identifying individuals who are at high risk for DM is potentially of significant benefit, so that preventive measures can be used in those individuals. Some studies have indicated that lifestyle interventions, offered to nondiabetic adults at high risk, could reduce or delay their conversion to DM. In previous studies, many risk factors have been associated with the incidence of DM in the Chinese population, like anthropometric measures, nutrition transition and changes in diet and lifestyle, smoking, genetic susceptibility, and so on [1]–[6]. Among these risk factors, anthropometric measures are simple and useful, which have been recommended for predicting future DM in many cliniccal practices [7], [8]. Recently, Krakauer NY et al [9] developed a new anthropometric measure (a body shape index, ABSI) based on WC that is approximately independent of height, weight and BMI, and the new anthropometric measure could predict premature mortality in the USA, even better than WC and BMI [9]. However, we don`t know whether the new anthropometric measure also could predict the new onset of DM in the Chinese population. Therefore, the main aims of the study were to assess the DM predictive power of ABSI based on the follow-up data over 15 years collected from a general Chinese population.

## Methods

The data were collected in 1992 and then again in 2007 from the same group of 711 participants in an urban community located in Chengdu, Sichuan province, China. In 1992, the method of non-random cluster sampling and cohort stratified random sampling was adopted, and medical professionals did a survey of cardiovascular diseases (CVD) risk factors in these participants, which were supported by a project from China's eighth national 5-year research plan (the Chinese multiprovincial cohort study). In 2007, we repeated a survey of these participants with the same methods, which was supported by megaprojects of science research for China's 11th 5-year plan (trends in the incidence of metabolic syndrome and integrated control in China). Other detailed information of these participants has been reported elsewhere [10]–[12]. Since 24 participants were diagnosed with diabetes in 1992, they were excluded from the analysis. Therefore, only 687 participants with complete data (male: 58.1%) were available and analysed. This study was approved by Ministry of Health of China, as well as by the Ethics Committee of West China Hospital of Sichuan University. All participants provided written informed consent.

WC at the end of a normal exhalation was measured to the midpoint between the lower border of the rib cage and the iliac crest. DM was defined by self-reported history or a fasting plasma glucose ≥7.0 mmol/L. ABSI was defined as WC/(BMI^2/3^height^1/2^), expressing WC and height in m [9].

Data are presented as means ± standard deviation (SD) for normal variables, or median + inter-quartile range for skewed variables. Comparisons between groups were performed by independent *t* test for normally distributed variables and by the nonparametric Mann-Whitney test for skewed variables. Interactions between categorical variables were evaluated with Pearson χ^2^ test. Correlations between different variables were used Pearson correlation analysis. Cox proportional hazards models were used to estimate hazard ratios (HRs) of incident DM associated with ABSI. Area under the receiver operating characteristic curve (AROC) was used to examine the discriminatory power of anthropometric measures for DM. Statistical significance was defined as *p*<0.05.

## Results

Of the 687 individuals without diabetes at baseline, 74 individuals were diagnosed with diabetes during a 15-year follow-up period (incidence 10.8%). In our study, the age distribution was 48.1±6.2 years, and the mean/variance of BMI, WC and ABSI were 23.4±2.8 kg/m^2^, 0.77±0.08 m and 0.0739±0.0044 m^11/6^kg^−2/3^, respectively. The 687 participants were divided into two groups according to the subsequent diabetic patients or not. In 1992, the demographic data showed age, BMI, WC, ABSI, fasting plasma glucose (FPG), total cholesterol (TC) and triglyceride (TG) were statistically significantly greater in the subsequent diabetic group, and HDL-C were statistically significantly lower (Table 1). Low-density lipoprotein cholesterol (LDL-C), sex and prevalence of hypertension did not differ between the two groups (Table 1).

**Table 1:** Baseline characteristics of our population according to diabetes status at follow-up.

Parameters	Subsequent diabetic patients (n = 74)	Subsequent non-diabetic patients (n = 613)	*p* value
Age (years)	49.8±5.7	47.9±6.2	0.013
Male sex	48 (64.9)	351 (57.3)	0.210
BMI (kg/m^2^)	25.1±3.3	23.2±2.6	<0.001
WC (m)	0.82±0.08	0.76±0.08	<0.001
ABSI (m^11/6^kg^−2/3^)	0.0757±0.0041	0.0737±0.0044	<0.001
SBP (mmHg)	119.5 (106.8, 129.3)	110.0 (104.0, 120.0)	0.021
DBP (mmHg)	75.7±9.6	72.0 (70.0, 80.0)	0.095
FPG (mmol/l)	4.6±0.8	4.0 (3.8, 4.7)	<0.001
TC (mmol/l)	4.7±0.7	4.3 (3.9, 5.0)	0.023
TG (mmol/l)	2.6±1.2	1.8 (1.5, 2.3)	<0.001
LDL-C (mmol/l)	2.3±0.9	2.3±0.8	0.776
HDL-C (mmol/l)	1.18±0.24	1.24 (1.06, 1.37)	0.007
Hypertension	16 (21.6)	88 (14.4)	0.099

Data are presented as means ± SD, or median (inter-quartile range), or number (percentage). BMI, body mass index; WC: waist circumference; ABSI: a body shape index; SBP, systolic blood pressure; DBP, diastolic blood pressure; TC, serum total cholesterol; LDL-C, low-density lipoprotein cholesterol; HDL-C, high-density lipoprotein cholesterol; TG, triglyceride; FPG, fasting plasma glucose.

Correlation coefficients of ABSI with WC, BMI, height and weight were 0.611 (*p*<0.001), −0.040 (*p* = 0.292), 0.283 (*p*<0.001) and 0.155 (*p*<0.001) respectively. Correlation coefficient of WC with BMI was 0.731 (*p*<0.001). After adjusting for age, sex, TC, LDL-C, HDL-C, FPG, TG and prevalence of hypertension, increasing the population ABSI by one SD would result in an increased HR of 41% (95%CI: 12%–77%), compared to 73% (95%CI: 37%–118%) for BMI and 96% (95%CI: 53%–150%) for WC. Since ABSI and BMI were uncorrelated in our study (r = −0.040), we presented an additional analysis with both ABSI and BMI included as predictors, to check whether the risk found by ABSI was complementary to that found by only using BMI. After adjusting for confounders, increasing the population ABSI by one SD would result in an increased HR of 53% (95%CI: 21%–95%), as well as 79% (95%CI: 43%–125%) for BMI. In Cox proportional hazard modeling, the relationship between hazard and continuous variables is most commonly estimated on the assumption that the logarithm of the hazard is a linear function of the variable. To quantify in a simple form the linear relationship between ABSI (BMI, WC) and log diabetic incidence, we carried out analyses where risk was computed separately for each quintile of the ABSI (BMI, WC), relative to the lowest quintile. Relative to the lowest quintile of ABSI (BMI, WC), the log DM hazard increased with increasing quintile (Table 2). On the other hand, WC had the best discriminatory power for DM (AROC: 0.701, 95%CI: 0.641–0.760), followed by BMI (AROC: 0.668, 95%CI: 0.601–0.734) and ABSI (AROC: 0.647, 95%CI: 0.581–0.713).

**Table 2:** Diabetic incidence and hazard ratios according to the quintile of ABSI, BMI and WC.

Quintile	Hazard ratios (95%CI)					
	ABSI	*p* value	BMI	*p* value	WC	*p* value
1 (lowest, reference)	1	N/A	1	N/A	1	N/A
2	1.518 (0.565−4.081)	0.408	1.986 (0.660−5.981)	0.222	2.541 (0.524−12.321)	0.247
3	1.458 (0.531−3.997)	0.464	2.593 (0.937−7.177)	0.067	5.083 (1.153−22.402)	0.032
4	1.713 (0.643−4.563)	0.282	2.279 (0.815−6.374)	0.116	6.538 (1.508−28.347)	0.012
5 (highest)	4.000 (1.621−9.871)	0.003	4.937(1.847−13.196)	0.001	7.628 (1.757−33.122)	0.007

ABSI: a body shape index; BMI, body mass index; WC: waist circumference. Results of Cox proportional hazard modeling for diabetic incidence with ABSI, BMI or WC quintiles taken as the predictors, adjusting for age, sex, TC, LDL-C, HDL-C, TG, FPG and prevalence of hypertension. Hazard ratios are relative to the lowest quintile in each case. The between-quintile cut points are 0.0702, 0.0726, 0.0749 and 0.0777 m^11/6^kg^−2/3^ for ABSI; 21.0, 22.5, 24.1 and 25.8 kg/m^2^ for BMI; 0.70, 0.74, 0.78 and 0.83 m for WC.

## Discussion

Our data showed that ABSI could predict the new onset of DM in the Chinese population independently. Since the confidence intervals for the HR coefficients and AROCs overlapped, the predictive ability of ABSI for DM was not better than WC and BMI. The three measures had similar predictive abilities for DM, not like the predictive ability for premature mortality in the published article [9]. Further studies about ethnic specificities of ABSI are needed and warranted.

Like other anthropometric measures [1], [2], [7], [8], ABSI could predict the new onset of DM independently. The possible human physiology was that high ABSI might correspond to a greater fraction of visceral fat compared to peripheral tissue at a given height and weight, and excess visceral fat has been associated with a variety of potentially adverse metabolic changes [9], which have been associated with the increased risk of DM [13]. Krakauer NY et al [9] thought the aforementioned human physiology also accounted for the substantial associations of ABSI with premature mortality. However, when we used ABSI to predict the new onset of DM, we didn`t get similar results like ABSI predicting premature mortality, which was better than WC and BMI [9]. We think ethnic differences might mainly cause the unexpected results. The new anthropometric measure (ABSI) based on a USA population sample from NHANES 1999–2004 (mainly including Mexicans, blacks and whites), however, the participants of the present study were Chinese. The new anthropometric measure mainly based on WC, and the imaging technologies have shown the amount of visceral adipose tissue was different between ethnic groups [9], [14]. Furthermore, ABSI showed little correlation with height, weight, or BMI (|r|<0.1) in the published article [9], but ABSI had some correlation with height or weight (r = 0.150–0.300) in our study, excepting BMI (|r|<0.1). On the other hand, the measurement of WC in our study is different from the published article [9]. Although in general the association of WC with health outcomes seems independent of the specified measurement protocol [15], we think this might still influence the results to some extent. To avoid the confounding factor, further studies should adopt the same measurement protocol. Finally, we should discuss an important issue. In our study, the mean/variance of BMI, WC and ABSI were lower in our study than in the publised article [9], and this might also be a possible reason for the seeming China-USA difference in its predictive ability. More cohorts are needed to delineate the limits of ABSI's utility.

There are some limitations. Firstly, the absence of oral glucose tolerance tests might miss some individuals who have developed DM. However, oral glucose tolerance tests for all participants were not feasible for pragmatic reasons and logistics. Secondly, there were no comparisons between different races.

In conclusion, the new anthropometric measure (ABSI) could predict the new onset of DM in the Chinese population independently. Since the confidence intervals for the HR coefficients and AROCs overlapped, the predictive ability of ABSI for DM was not better than WC and BMI. The three measures had similar predictive abilities for DM, not like the predictive ability for premature mortality in the published article [9]. Further studies about ethnic specificities of ABSI are needed and warranted.
